# Adult neurogenesis in intellectual disabilities

**DOI:** 10.18632/oncotarget.18476

**Published:** 2017-06-14

**Authors:** Manuela Allegra, Matteo Caleo

**Affiliations:** CNR Neuroscience Institute, G. Moruzzi, Pisa, Italy

**Keywords:** Oligophrenin-1, rho GTPase, fasudil, hippocampus

In mammals, new neurons are continuously produced in two specific neurogenic niches, the subventricular zone (SVZ) and the subgranular zone (SGZ) in the dentate gyrus (DG) of the hippocampus. Adult neurogenesis recapitulates some of the key events of brain development including proliferation of neuronal progenitors, migration and morphological maturation of newly generated cells, and functional integration into brain networks. In the SGZ, neural progenitors differentiate into dentate granule cells (DGCs), which elaborate their dendritic arbor into the molecular layer and extend axons to innervate target cells in the CA3 region. To be integrated, adult born neurons start to form afferent and efferent connections, and this process of synaptogenesis is under the control of several local and environmental factors. During maturation of their physiology and connectivity, the newborn neurons exhibit increased excitability and enhanced synaptic plasticity. Due to their role in hippocampal plasticity, the adult born cells may affect hippocampus-dependent learning and memory. Recent studies have highlighted a crucial role for adult neurogenesis in the acquisition/forgetting of memories and in the discrimination of spatial and contextual information [1].

It is well recognized that adult neurogenesis is affected in various neurological diseases associated with cognitive impairments, such as intellectual disabilities (ID), including Rett, Fragile X and Down syndromes. ID is a complex disease of the central nervous system (CNS) whose pathophysiology is not completely understood and no effective cures are available to date.

Oligophrenin-1 (Ophn1) gene, located on X chromosome, encodes for a RhoGTPase-activating protein (RhoGAP) whose mutations are involved in the etiology of X-linked intellectual disability (XLID). Ophn1 is expressed in the whole brain during development and in adulthood. At the cellular level, Ophn1 is expressed in both glial and neuronal cells where it colocalizes with actin. Due to its interaction with actin, Ophn1 participates in several neuronal developmental processes, including dendrite and axon growth, axon guidance, synapse formation and cell migration [2]. Ophn1 has both a pre- and post-synaptic localization, influencing dendritic spine morphogenesis and synaptic function and plasticity [3, 4]. At the electrophysiological level, the loss of function of Ophn1 leads to impairments of both excitatory and inhibitory synaptic transmission. Ophn1 is a key negative regulator of RhoGTPases, a family of molecules (including RhoA, Rac and Cdc42) that orchestrate various pathways and transduce signals from the extracellular environment to the actin cytoskeleton.

In a recent manuscript [5], we have examined the impact of Ophn1 deficiency on neuronal maturation by following the process of adult neurogenesis in the DG. Using Ophn1 knock-out (KO) mice [4], we found significant deficits in the morphological maturation and survival of newborn neurons in the DG. Specifically, Ophn1-deficient newborn neurons showed alterations in axon extension and dendritic maturation [5]. In particular, the aberrant axon formation in Ophn1 KO animals, leading to altered synaptic connectivity, supports the hypothesis that IDs are characterized by circuit impairments due to morphological or wiring alterations during neuronal development. Moreover, we also found an immature phenotype of dendritic spines, consistent with recent findings demonstrating that thin, filopodia-like dendritic spines are increased in animals lacking Ophn1 [6].

Ophn1 loss of function results in persistent activation of RhoGTPases and consequent hyperstimulation of the RhoA/ROCK pathway and high levels of PKA activity. Based on previous data [7], we used the ROCK/PKA inhibitor fasudil, a drug already approved for clinical use in Japan and China, as a pharmacological approach to treat the impairments associated with Ophn1 deficiency. We found that chronic fasudil treatment reversed at least some of the pathological alterations of adult hippocampal neurogenesis in Ophn1 KO mice. While the deficits in axon extension were not affected by the treatment, fasudil completely rescued the dendritic spine deficit and enhanced the long-term survival of newly generated neurons. Similar rescue effects have been reported in the context of olfactory bulb neurogenesis [6].

One fundamental question in these studies is whether the normalization of adult neurogenesis improves cognitive functions. Our data [5] and those of Redolfi et al. [6] clearly demonstrate that counteracting the unchecked up-regulation of ROCK/PKA signalling by fasudil treatment rescues adult neurogenesis. Parallel experiments have shown that fasudil treatment normalizes performance in specific hippocampus-related behavioural tasks [8], highlighting the idea that adult neurogenesis could be a potential pharmacological target for cognitive impairments associated with ID.

Altogether, the data described above indicate that adult neurogenesis allows a precise dissection of alterations of neuronal maturation in IDs, and the experimental validation of novel therapeutic strategies.
